# Supplementary material for: Distinct compositions and functions of circulating microbial DNA in the peripheral blood compared to fecal microbial DNA in healthy individuals
Source: mSystems. 2024 Mar 1;9(3):e00008-24. doi: 10.1128/msystems.00008-24 (PMC10949464; doi:10.1128/msystems.00008-24)
Supplement: Table S4 — Experimental and control group settings [file msystems.00008-24-s0004.docx]

| Group | Detection substance |
| --- | --- |
| Blood | Plasm |
| Fecal | Fecal |
| Urine | Urine |
| Negative controls | Microorganisms from sample collection sites |
| Blank controls | Nuclease-free water |
| Extraction controls | Nuclease-free water |
| Amplification controls | Nuclease-free water |

Supplement Table 4：Experimental and control group settings
